# Genome-wide methylation and expression profiling identifies promoter characteristics affecting demethylation-induced gene up-regulation in melanoma

**DOI:** 10.1186/1755-8794-3-4

**Published:** 2010-02-09

**Authors:** Jill C Rubinstein, Nam Tran, Shuangge Ma, Ruth Halaban, Michael Krauthammer

**Affiliations:** 1 Interdepartmental Program in Computational Biology & Bioinformatics, Yale University, New Haven, CT 06520, USA; 2 Department of Pathology, Yale University, New Haven, CT 06520, USA; 3 School of Public Health, Yale University, New Haven, CT 06520, USA; 4 Department of Dermatology, Yale University, New Haven, CT 06520, USA

## Abstract

**Background:**

Abberant DNA methylation at CpG dinucleotides represents a common mechanism of transcriptional silencing in cancer. Since CpG methylation is a reversible event, tumor supressor genes that have undergone silencing through this mechanism represent promising targets for epigenetically active anti-cancer therapy. The cytosine analog 5-aza-2'-deoxycytidine (decitabine) induces genomic hypomethylation by inhibiting DNA methyltransferase, and is an example of an epigenetic agent that is thought to act by up-regulating silenced genes.

**Methods:**

It is unclear why decitabine causes some silenced loci to re-express, while others remain inactive. By applying data-mining techniques to large-scale datasets, we attempted to elucidate the qualities of promoter regions that define susceptibility to the drug's action. Our experimental data, derived from melanoma cell strains, consist of genome-wide gene expression data before and after treatment with decitabine, as well as genome-wide data on un-treated promoter methylation status, and validation of specific genes by bisulfite sequencing.

**Results:**

We show that the combination of promoter CpG content and methylation level informs the ability of decitabine treatment to up-regulate gene expression. Promoters with high methylation levels and intermediate CpG content appear most susceptible to up-regulation by decitabine, whereas few of those highly methylated promoters with high CpG content are up-regulated. For promoters with low methylation levels, those with high CpG content are more likely to be up-regulated, whereas those with low CpG content are underrepresented among up-regulated genes.

**Conclusions:**

Clinically, elucidating the patterns of action of decitabine could aid in predicting the likelihood of up-regulating epigenetically silenced tumor suppressor genes and others from pathways involved with tumor biology. As a first step toward an eventual translational application, we build a classifier to predict gene up-regulation based on promoter methylation and CpG content, which achieves a performance of 0.77 AUC.

## Background

Epigenetic abnormalities, including global losses and local gains in methylation, have been observed in many types of cancer, including melanoma [1-3]. It is thought that, while global hypomethylation may induce genomic instability early in cellular transformation, localized hypermethylation may promote tumorigenesis through silencing of tumor suppressor genes [4,5]. Some genomic loci are more susceptible to such hypermethylation than others, and significant progress has been made in predicting which CpG islands will be subject to methylation on the basis of sequence motifs [6,7].

A well-established relationship exists between promoter methylation and transcriptional repression [8-10]. There are two popular models for this phenomenon, the first positing that the methyl groups directly block the binding of transcription factors [11] and the second citing the role of methyl-binding proteins that recruit transcriptional repressors to the methylated sites [12,13]. Recently, Koga et al. used MeDIP combined with promoter tiling microarrays to evaluate genome-wide methylation levels and transcriptional regulation in a set of melanoma cell strains [14]. Their analysis confirmed that, for promoters containing a minimum number of CpG dinucleotides, increased methylation caused decreased expression.

Recognition of the importance of epigenetic silencing in tumor biology has led to exploration of the therapeutic potential of demethylating agents, such as the DNA methyltransferase inhibitor 5-aza-2'-deoxycytidine (decitabine). The drug received FDA approval for the treatment of myelodysplastic syndrome in 2004 and is currently the subject of clinical trials exploring its utility in treating a variety of solid tumors. The ability of these agents to up-regulate the expression of aberrantly suppressed genes has been demonstrated in several studies, resulting in lists of individual candidate targets for demethylation-induced up-regulation [15-18]. Similar studies have employed RNAi for DNMT knockdown toward the same end [19]. While these efforts are providing valuable insight into the specific genes and gene pathways that may be targetable through epigenetic manipulation, it is still unclear why decitabine causes some silenced loci to become up-regulated, while others remain inactive. We therefore embarked on systematic, genome-wide studies exploring molecular characteristics of decitabine-responsive genes.

Using microarray analysis of both gene expression and promoter methylation, we sought to identify promoter characteristics that predict the likelihood of response to decitabine treatment. We began by stratifying the promoter regions on the basis of CpG content and pre-decitabine methylation level. We then tested for enrichment of up-regulated genes in each of the resulting promoter categories (i.e., different CpG content and methylation level combinations). Using logistic regression and ten-fold cross-validation, we trained and tested a classifier that predicts the likelihood of decitabine-induced up-regulation on the basis of promoter category.

## Results

### Decitabine-induced up-regulation varies by promoter methylation level and CpG content

Using the melanoma methylation dataset from Koga et al. [14], combined with gene expression measurements on the same cell strains (Table 1), treated and untreated with decitabine [20], we were able to systematically investigate differential gene expression, as well as the relationship to basal methylation level and CpG content, in response to relatively low doses of the drug.

**Table 1:** Melanoma cell strains.

Melanoma cell line	Gender/age	Stage/site
YURIF	M/53	IV/soft-tissue metastasis, right thigh

WW165	F/62	Primary melanoma, 2.25 mm

YUGEN8	F/44	IV/brain metastasis

YUSIT1	M/67	Metastatic melanoma

YULAC	F/66	IV/soft-tissue metastasis, neck

YUSAC	M/57	IV/soft-tissue metastasis, left neck

YUMAC	M/68	IV, soft-tissue metastasis, right thigh

**Figure 1:**
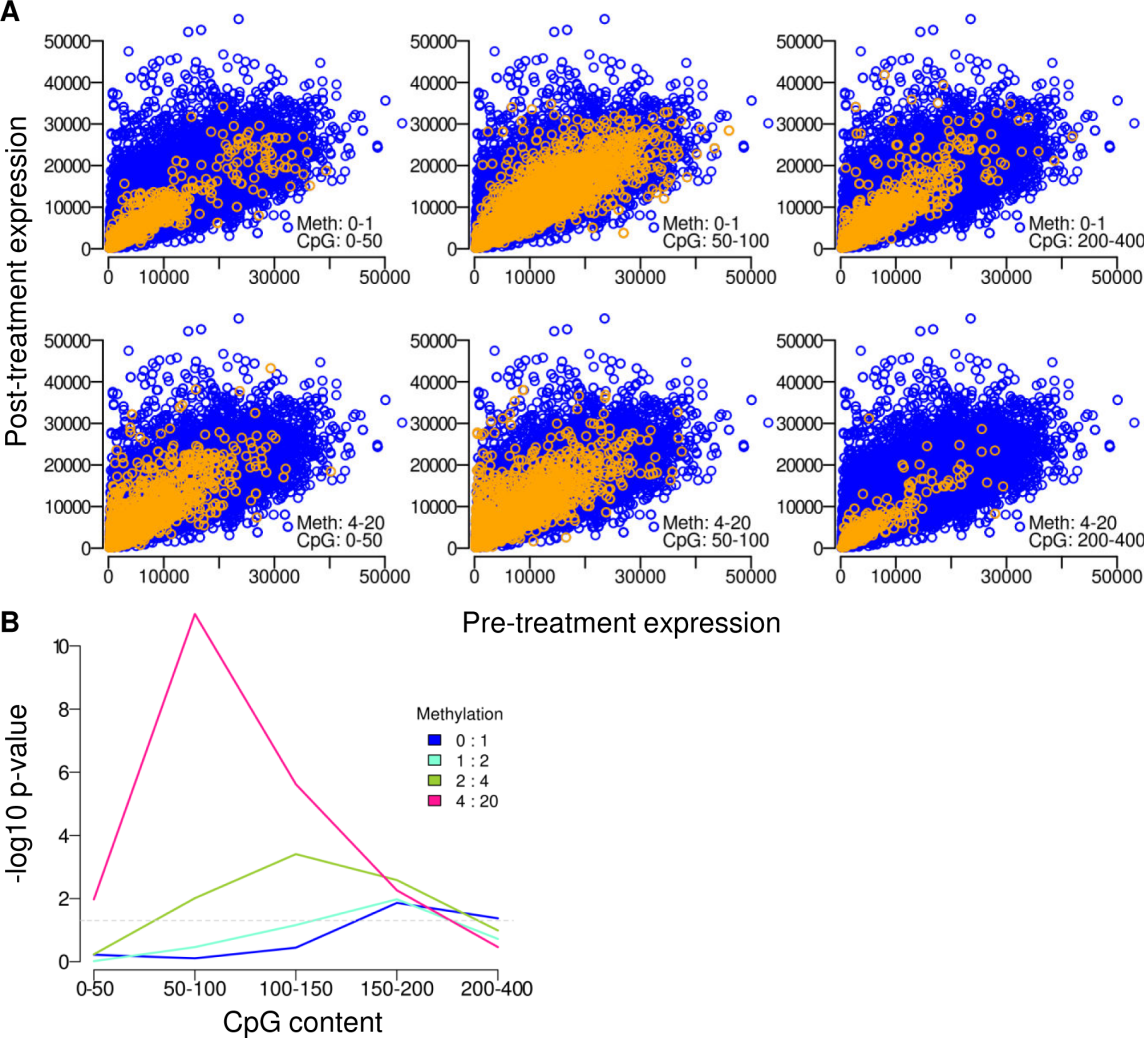
**Decitabine effect as related to promoter methylation level and CpG content**. **Panel A**. Genes are stratified by promoter CpG content (increasing from left to right) and methylation level (low methylation in top row, high methylation in bottom row). Plotting post-treatment expression level as a function of basal levels demonstrates the distribution of promoters in each bin (orange dots) in the context of all promoters (blue dots). **Panel B**. Summary of -log p-value from Wilcoxon signed-rank test assessing the likelihood that basal expression < post-treatment expression. The dotted line corresponds to -log10 of 0.05. The promoter category with the most significant trend toward up-regulated genes has highly methylated promoters with intermediate CpG content. As methylation level decreases, up-regulation is apparent in progressively more CpG dense promoters.

Stratifying promoters by methylation level and CpG content, we first assessed the effects of decitabine on absolute expression level as a function of un-treated level. Figure 1A shows the un-treated vs treated expression levels for promoters with low levels of methylation (top row) vs. those that are highly methylated (bottom row) across a range of promoter CpG contents (increasing from left to right). For each promoter category we use the non-parametric Wilcoxon signed-rank test to assess the likelihood that expression levels are lower before treatment than after. Figure 1B shows the -log10 of the p-values resulting from this test, demonstrating that the most significant up-regulation occurs in highly methylated genes with lower CpG content. As methylation level decreases, the likelihood of significant up-regulation occurs at progressively higher CpG content.

### Defining decitabine-responsiveness

Defining the effect of decitabine on transcription in terms of the fold-change in expression before and after treatment (as is often done for differential expression) might be misleading, resulting in a biased definition of up-regulated genes. Genes with low basal expression require a lesser absolute increase to meet a 2-fold up-regulation threshold after treatment than do genes with high basal expression. Also, genes with very low basal expression might show a two-fold increase in response to decitabine but still have biologically insignificant amounts of gene expression. Furthermore, it is important to note that the set of genes with low basal expression values are significantly enriched for low CpG-content promoters, whereas genes with high CpG-content promoters generally have greater baseline expression levels [21]. An up-regulation threshold that favors genes with low starting expression would therefore bias the set of decitabine-responsive genes to include a greater proportion of low CpG-content promoters. Given that our initial observation suggests that promoter CpG content is an important factor in decitabine-responsiveness, we were particularly interested in addressing this bias. To do so, in addition to a 2-fold increase in expression, we require a minimum delta expression of 5,000 units in order to consider a gene up-regulated. Additional file 1 contains the list of refseq IDs for each promoter bin, as well as an indication of which are up-regulated.

**Figure 2:**
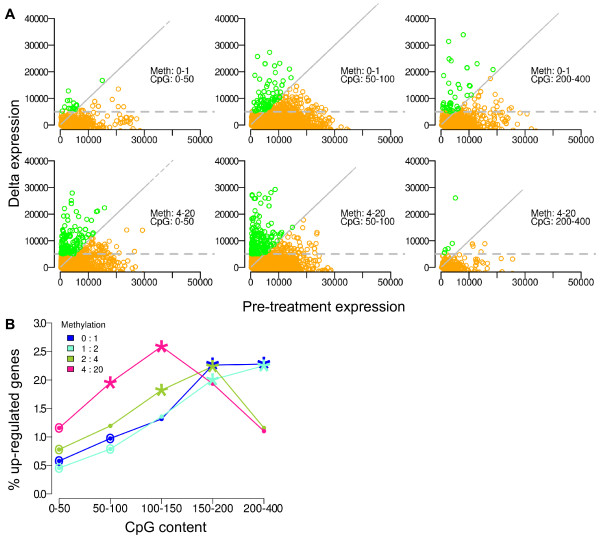
**Defining decitabine-response by fold-change expression and delta expression**. **Panel A**. Genes are stratified by promoter CpG content (increasing from left to right) and methylation level (low methylation in top row, high methylation in bottom row). Delta expression is plotted as a function of basal expression level. Upregulated genes are defined as those with >= 2-fold expression increase (left of diagonal line) and delta expression >= 5,000 (above horizontal line). **Panel B**. Percent of upregulated genes per CpG and methylation bin, with maximal up-regulation observed at high methylation and intermediate CpG content. Stars and circles indicate promoter categories with statistically significant enrichment or depletion in the number of up-regulated genes, respectively.

Figure 2A shows delta expression vs. basal expression, again stratified by promoter methylation level and CpG content. Genes to the left of the diagonal and above the horizontal line are those that meet the criteria of up-regulation. In total, 2,265 genes from all genes pooled from 7 cell strains met the criteria for up-regulation. Again, we observe that certain CpG content/methylation bins are enriched for induced genes. On a gross scale, promoters with low levels of methylation (top row), have peak numbers of up-regulated genes in the more CpG-dense promoter bins. Whereas, for highly methylated promoters, more up-regulation is observed in the intermediate CpG content bins. Figure 2B summarizes the percent of up-regulated genes by bin, again demonstrating that the greatest subset of induced genes lies within the highly methylated promoters with intermediate CpG content. As methylation level decreases, the peak amount of up-regulation occurs in progressivley more CpG-dense promoter categories.

We next employed the hypergeometric distribution to test, for each promoter category, whether the number of up-regulated genes is significantly different than would be expected by chance. Those promoter categories significantly enriched or depleted in up-regulated genes are denoted in figure 2B with a star or a circle, respectively, and the pattern confirms our previously observed trend.

We also investigated whether gene upregulation is explained by factors other than promoter methylation and CpG density. Using the GSEA method over gene expression measurements in the YUMAC strain, we tested for Gene Ontology (GO), KEGG pathway and motif gene set enrichments among the upregulated genes. Our results show that none of the gene sets were enriched at the FDR level of 0.05, indicating that gene upregulation is not dominated by activation of a particular cellular process, or regulation by a transcription factor.

### Decitabine-responsive MCF-7 genes

**Figure 3:**
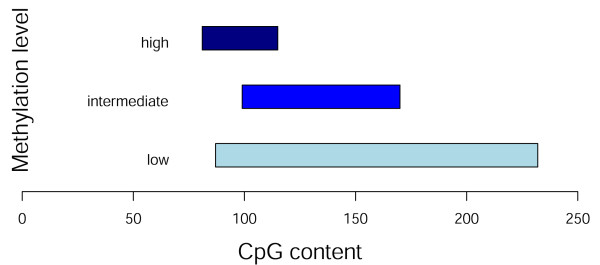
**Promoter methylation and CpG content of decitabine-responsive genes in MCF-7 cell line**. Of eighteen decitabine-induced genes from the MCF-7 breast cancer cell line, three are highly methylated, five have intermediate methylation levels, and ten have low levels of methylation. Highly methylated, decitabine-responsive genes have lower promoter CpG content. With increasing levels of methylation, CpG content of responsive genes increases. For extremely CpG-dense promoters, only those with low methylation levels are up-regulated. These observations are in accord with the melanoma data.

To investigate whether the observed trend was present in an independent cell line, we obtained publicly available data. These included expression data in response to decitabine for the MCF-7 breast cancer cell line from the BROAD connectivity map database [22], and promoter methylation data for the MCF-7 cell line [23]. We analyzed those genes with at least a 2-fold increase in expression following treatment, identifying 18 genes for which we could also obtain methylation data (see Additional file 1). Among these, three of the promoters were highly methylated, five showed intermediate levels of methylation, and ten had low methylation levels. All three of the decitabine-responsive genes with highly methylated promoters had low to intermediate CpG content. For intermediately methylated promoters, the range of CpG content shifted toward greater density. Furthermore, extremely CpG dense promoters were up-regulated only when accompanied by low basal methylation levels. Figure 3 summarizes these findings, all of which are consistent with our observations in melanoma cell strains.

While this independent dataset is limited in size, the overall trend is consistent with our findings and we found no evidence to contradict our hypothesis. Importantly, the MCF-7 cell lines were treated with a low dose decitabine (100 nM) that is comparable to the 200 nM used in the melanoma experiments. One caveat to the comparison is that the decitabine-response data for the MCF-7 cell line is in the form of fold-change following treatment; we can therefore not account for potential bias introduced by basal expression levels (discussed above).

### Predicting decitabine response

**Figure 4:**
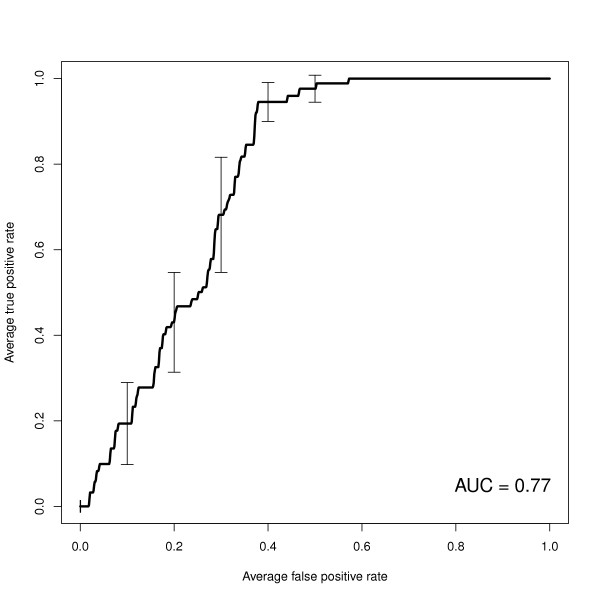
**Predicting decitabine-responsive genes using logistic regression**. Receiver operating curve for predicting decitabine response on the basis of promoter CpG content and methylation level. Models were trained and tested on the YUMAC cell line using ten-fold cross-validation. The plot shows the mean across ten runs, with 2*standard error bars. The area under the curve (AUC) is 77%, compared to 72% and 63%, respectively, for models built on promoter methylation status or CpG content alone.

To test whether the observed trend in promoter characteristics could be used to build a rudimentary classifier, we trained a logistic regression model to predict individual gene reponse on the basis of promoter methylation and CpG density. As the clinical goal is to induce expression of silenced tumor supressor genes, we began by taking the subset of genes with low basal expression. Using the YUMAC cell strain, which displayed the greatest absolute number of decitabine-responsive genes, we trained and tested the model using methylation and CpG content across the promoter, from 2,200 basepairs upstream to 500 basepairs downstream of the transcription start site. Figure 4 shows the average of the receiver operating curves, including error bars for 2 × standard error, using ten-fold cross-validation.

We found that the combination of promoter methylation and CpG content across the entire promoter has an area under the ROC (AUC) of 77%. The predictive power is superior compared to either methylation status or CpG content alone (AUC 72% and 63%, respectively).

To investigate the impact of more localized measures of methylation, we next repeated the above analysis for each of six bins at varying distances from the transcription start site (Table 2). The classifier performance was uniform across the bins, and was lower than using the cumulative methylation and CpG density.

**Table 2:** Performance of predictive models of up-regulation.

Distance from TSS	AUC (%)
-2200 to +500	77.0

-2200 to -1500	68.7

-1500 to -1000	68.2

-1000 to -500	70.9

-500 to -200	70.3

-200 to +100	69.1

+100 to +500	71.4

AUC by promoter location after 10-fold cross-validation.

To further strengthen confidence in the model, we performed 500 permutations of the class label, training and testing the model with ten-fold cross-validation each time. The mean AUC for these 500 permutations was 50.4%, as would be expected of a model with no predictive power. This analysis provides further evidence that the trend observed in promoter characteristics of up-regulated genes was not due to chance alone.

## Discussion and Conclusion

The ability to re-activate genes that have been epigenetically silenced in cancer could prove a powerful adjunct to existing chemotherapeutics, yet not all methylated genes undergo up-regulation following demethylating treatment. We detect patterns of promoter characteristics that influence susceptibility to decitabine treatment in a group of melanoma cell strains.

Promoters with a high degree of methylation and an intermediate CpG content appear most susceptible to decitabine-induced up-regulation. For these highly methylated promoters, as the CpG content increases, the efficacy of the drug appears to decrease. We conclude that, in CpG-dense promoters with high levels of methylation, the large absolute number of methyl groups overwhelms the ability of the drug to demethylate sufficiently to allow increased expression.

In contrast, for genes with low levels of methylation, the drug's effects become more pronounced as CpG content increases. We theorize that, in promoters that are sufficiently CpG-dense, those with lesser levels of methylation might still contain an absolute number of methyl groups large enough to constitute a viable target for demethylation-induced expression. Silent promoters that are CpG-sparse and have low levels of methylation are likely repressed by regulatory mechanisms that are not responsive to demethylation.

In addition to examining promoter CpG content and methylation level, we have performed initial analyses (data not shown) to suggest that the presence of DNase hypersensitivity sites, reflecting an open chromatin state, also plays a role in determining a promoter's susceptibility to demethylation-induced up-regulation of expression. Further studies using chromatin structure data, as well as more refined analysis of promoter sequence features, will likely improve upon the classifier's predictive power.

In summary, we identified a trend in promoter characteristics that correlates with the likelihood of response to decitabine in a set of melanoma cell strains, and used this trend to build a computational classifier to predict response to treatment. Further study using higher resolution assessment of methylation, as well as integration with genome-wide promoter architecture data (such as DNase hypersensitivity and histone modification) is needed to decipher in more detail the regulatory forces causing gene silencing and the likelihood of up-regulating key tumor supressor genes using drugs that target DNA methylation.

## Methods

Methods for cell isolation, culture, drug treatment, MeDIP, and methylation and expression-profiling were previously reported [14,20]. Briefly, methylated DNA was enriched using the MeDIP approach followed by hybridization to genomic promoter tiling arrays (NimbleGen C426-00-01) containing 390,000 probes. Standard normalization methods for two-channel arrays were applied, and relative methylation levels were determined using the MEDME bioconductor library [24]. Approximately 20-30 million cells were used for each mRNA extraction. Cells were treated with low-dose (200 nM) decitabine for two days, followed by one day recovery before total RNA extraction. Gene expression data was derived from NimbleGen human whole genome expression microarrays (array 2005_04-20_Human_60 mer_1in2) containing 380,000 probes with an average of 11 probes per refseq, located throughout the gene. Probe measurements were then averaged for each refseq. The same chip was hybridizied with differentially labeled, polyA-selected cDNA from decitabine-treated and untreated cells, experiments were repeated with dye swapping. Data was captured and processed by NimbleGen Systems Iceland LLC. Normalization within arrays was performed with Loess-based methods to correct for biases due to labeling with different dyes on the two microarray channels and to correct for spatial artifacts. As such, M and A values were determined where M describes the amount of differential expression (M = log2(cy5/cy3)) and A associates M with the magnitude of overall expression (A = (log2cy5+log2cy3)/2). Normalization between arrays was performed via quantile-based normalization. mRNA RefSeqs were mapped to the genome and those with < 96% sequence identity, as well as those that mapped to more than two genomic loci, were discarded. Analysis of the data revealed upregulation of 292 common genes across the cell lines after Decitabine treatment, and the treatment effect on demethylation was validated in selected strongly upregulated genes, such as CDKN1A and TGFBI.

For our experiments on decitabine-induced gene upregulation, we pooled information on gene promoter methylation level, CpG density, as well as differential gene expression from 7 cell strains. Data pooling yielded measurements for 22,824 promoters per cell strain, for a total of 159,768 data triplets (methylation, CpG density and differential gene expression).

For each promoter, the sequence from 2,200 basepairs upstream to 500 basepairs downstream of the transcription start site (TSS) was analyzed for CpG content, and five equal categories defined. Methylation levels for these promoters were previously reported for each of six bins spanning the same 2,700 basepairs around the TSS [14]. In order to obtain a single methylation measurement for each promoter, we used the sum of these six values and divided the results into four categories such that each bin contains roughly the same number of promoters (~40,000). Basal and decitabine-induced expression values represent the mean of two replicate experiments for each promoter. Promoters that demonstrate a two-fold increase in expression (post-treatment/pre-treatment expression >= 2) as well as an absolute expression increase of at least 5,000 units are labeled as up-regulated.

For each combination of CpG content and methylation level, the non-parametric wilcoxon signed-rank test was employed to compare post-decitabine to pre-decitabine expression levels. For this analysis we used the R stats module wilcox.test function.

For each combination of CpG content and methylation level, the significance of the difference between the observed number of up-regulated genes and the number expected by chance alone (total number in the bin multiplied by the fraction of all genes that undergo up-regulation) is calculated from the hypergeometric distribution using the R stats module dhyper function with the number up-regulated in the CpG/methylation bin, total number of up-regulated promoters, total number not up-regulated, and number in the CpG/methylation bin.

We used the GSEA program http://www.broadinstitute.org/gsea/ to analyze our expression data for enriched Gene Ontology and motif gene sets. We uploaded the gct and cls files corresponding to our data from the YUMAC cell strain, and set the *Metric for ranking gene*s to *Ratio_of_Classes*, and the *Permutation type *to *gene_set*. All other parameters were set to default.

Expression response data following decitabine treatment on the MCF-7 breast cancer cell line was downloaded from the BROAD Institute Connectivity Map [22]. Methylation data for the MCF-7 cell line was downloaded from the supplementary material from a study by Li et al. that used a modified methylation-specific digital karyotyping for genome-wide methylation profiling of two breast cancer cell lines [23]. Methylation levels were in the form of the number of sequencing reads per fragment. Using the 90th quantiles from the MCF-7 and melanoma datasets, the MCF-7 methylation levels were scaled so that the distribution of values between the two datasets occupied an equivalent range. MCF-7 promoters were then categorized as having either low (0-1), intermediate (1-6), or high (>6) levels of methylation.

A computational model of decitabine-response was built using the generalized linear model for logistic regression. This was implemented using the R stats module glm function with the following arguments: formula = upregulated ~promoter methylation + promoter CpG content; family = gaussian; method = glm.fit (iteratively weighted least squares). Briefly, data from the YUMAC cell line was filtered for genes with pre-treatment expression levels below 700 units (app. 40% of the data). For each promoter bin, and for the region as a whole, the model was then trained and tested using ten-fold cross-validation, receiver operating curves were generated and AUCs calculated using the ROCR package [25]. The class labels were then permuted 500 times, the model trained and tested for each permutation, and the mean AUC calculated.

**Gene expression and promoter methylation data have been uploaded to GEO (Accession: GSE13706) and ArrayExpress (Accession: E-MTAB-185)**.

## Competing interests

The authors declare that they have no competing interests.

## Authors' contributions

JCR lead the study design, carried out the computational studies, and drafted the manuscript. NT participated in the design of the study. SM provided statistical support. RH provided the experimental results. MOK conceived the study and participated in its design and coordination. All authors read and approved the final manuscript.

## Pre-publication history

The pre-publication history for this paper can be accessed here:

http://www.biomedcentral.com/1755-8794/3/4/prepub

## Supplementary Material

Additional file 1 **Genes identifiers**. The file GenesByBin.txt contains refseq ID, name of the cell line, and class assignment (up-regulation by Decitabine) for each gene analyzed in the study. The file is organized by promoter category as defined in the text. The file also contains identifiers for the MCF-7 genes included in the analysis. Click here for file
